# Commentary response: Addressing the scientific as well as the social‐inequalities questions ‐ Response to the commentary of Newlove‐Delgado (2021)

**DOI:** 10.1111/jcv2.12015

**Published:** 2021-05-19

**Authors:** Nicola Wright, Jonathan Hill, Helen Sharp, Andrew Pickles

**Affiliations:** ^1^ Department of Biostatistics & Health Informatics Institute of Psychiatry King's College London London UK; ^2^ Department of Child and Adolescent Psychiatry School of Psychology and Clinical Language Sciences University of Reading Reading UK; ^3^ Department of Perinatal and Child and Adolescent Clinical Psychology Institute of Life and Human Sciences University of Liverpool Liverpool UK; ^4^ Department of Biostatistics & Health Informatics Institute of Psychiatry King's College London London UK

Recent reviews of the scientific challenges posed by the study of children's mental health during COVID‐19 have highlighted the problems in establishing the effect and the risks arising from sample biases. In order to strengthen inference about causal processes, ‘Existing longitudinal cohort studies, with measures already taken before lockdown and assessment of postlockdown mental health, will take us some of the way by providing important information about temporal sequencing of the exposure and its ‘effect’. (Sonuga‐Barke 2021). In order to deal with selection biases associated with the psychosocial processes we seek to study, funders and researchers should, ‘…gather, timely, high‐quality population mental health data that represent the true need arising from the pandemic’ (Pierce et al, 2020).

Our paper reports the findings obtained by interrupting data collection during the 15th assessment wave of a general population sample recruited during pregnancy, and assessing again three months later, with 89% retention. This provides a design almost as strong as a randomised control trial, so the study findings can be readily attributed to a COVID‐19 pandemic effect, conducted with a well‐characterised sample and known sources of bias. It thus addresses both of these scientific challenges. By virtue of the narrow age range of the participating children, in early adolescence, it also examines impact over a crucial period for the emergence of psychopathology. Unlike the other studies considered in the commentary, measurement from pregnancy also allows us to examine the interplay between long‐term vulnerability and COVID‐19 impact.

These points provide important context for the comment by Newlove‐Delgado (2021), ‘…this study, and many of the others described here, publish data collected 9–12 months ago. This is a blink of the eye in research and publishing timescales, but in the context of the pandemic feels more like looking through a telescope at light from stars that might no longer exist.’ This could unfortunately be interpreted to mean that our findings of large effects on depression and behavioural problems in young adolescents, and our examination of the role of prior vulnerability, should be consigned to the outer reaches of the developmental cosmos. Rather, they are relevant here and now. Only continued follow‐up of studies such as ours, with strong claims on causality, generalisability, and high retention, can provide high quality longitudinal data on persistence and recovery, and on what creates vulnerability and confers resilience in the context of the COVID‐19 pandemic over time.

## CONFLICT OF INTERESTS

No conflicts of interest to declare.

## AUTHOR CONTRIBUTIONS

We all equally wrote and reviewed the response.

## Data Availability

Due to ethical constraints, supporting data cannot be made openly available. Supporting data are available to bona fide researchers on approval of an application for access. Further information about the data and conditions for access are available at the University of Liverpool Research Data Catalogue: DOI: 10.17638/datacat.liverpool.ac.uk/564.
